# Spatial behavior and habitat use in widely separated breeding and wintering distributions across three species of long‐distance migrant* Phylloscopus* warblers

**DOI:** 10.1002/ece3.5226

**Published:** 2019-05-24

**Authors:** Mathilde Lerche‐Jørgensen, John W. Mallord, Mikkel Willemoes, Christopher J. Orsman, Japheth T. Roberts, Roger Q. Skeen, Daniel P. Eskildsen, Volker Salewski, Anders P. Tøttrup, Kasper Thorup

**Affiliations:** ^1^ Center for Macroecology, Evolution and Climate, Natural History Museum of Denmark University of Copenhagen Copenhagen Denmark; ^2^ RSPB Centre for Conservation Science, Royal Society for the Protection of Birds The Lodge Sandy UK; ^3^ Ghana Wildlife Society Efua Sutherland Children's Park Accra Ghana; ^4^ Birdlife Denmark Copenhagen V Denmark; ^5^ Michael‐Otto‐Institut im NABU Bergenhusen Germany

**Keywords:** Afro‐Palearctic migrants, home range, *Phylloscopus*, radio tracking

## Abstract

**Aim:**

To investigate the ecological relationship between breeding and wintering in specialist and generalist long‐distance migratory species, and the links between densities and range sizes.

**Location:**

Denmark, Senegal and Ghana.

**Methods:**

We use radio tracking to study spatial behavior and habitat use in three morphologically and ecologically similar and closely related *Phylloscopus* species on their widely separated breeding and wintering distributions. During wintering and breeding, willow warblers *P. trochilus* (winter: *n* = 9, breeding: *n* = 13), chiffchaffs *P. collybita* (*n* = 11, *n* = 7), and wood warblers *P. sibilatrix* (*n* = 17, *n* = 14) were tracked.

**Results:**

Willow warblers *P. trochilus* increased home range sizes in winter, whereas it was similar in chiffchaffs *P. collybita* and wood warblers *P. sibilatrix*, in both seasons. Home ranges overlapped more in winter than in the breeding season. In winter, home range overlap was similar among species but larger overlap during breeding was indicated for willow warblers. Tree cover was unrelated to home range size but significantly higher in breeding than in winter in all species. However, whereas willow warblers and wood warblers maintained some degree of tall tree cover inside their home ranges in winter, chiffchaffs changed from more than 80% to <1% tree cover, indicating a niche shift.

**Main conclusions:**

Individuals of all three species showed changes between breeding and wintering areas in spatial behavior and habitat availability, with larger overlap in winter. The differences in patterns were potentially related to being generalist (willow warbler) or specialist (chiffchaff and wood warbler). These ecological relationships are important for the conservation of migrants and for understanding the link between breeding and wintering distributions and ecology.

## 1 INTRODUCTION

Billions of birds move from breeding at higher to wintering at lower latitudes tracking resource abundance across seasons (Newton, 2008). Long‐distance migrants are declining at a faster rate than resident or short‐distance migrant species (Vickery et al., 2014), yet the drivers of these declines remain largely unknown (Bairlein, 2016; Vickery et al., 2014). Conservation of migrating species poses a special challenge as populations can potentially be adversely affected by habitat and climate change at any site used during the annual cycle (Runge, Martin, Possingham, Willis, & Fuller, 2014). Therefore, it is vital for conservation of migrants to understand dependencies and link biology across seasons and sites (Runge et al., 2014).

Space and habitat use likely depends on life‐history stage (breeding or wintering) and species‐specific factors. On the breeding grounds, home range or territory size reflects the area needed to provide sufficient food to raise young (Gill, 1989; Scott, 2010). If habitat quality is similar, the area needed in the breeding season, when they provide food for chicks, is expected to be larger than the area needed to provide food enough for themselves. However, it is potentially constrained by having to be close to the nesting site and defend a territory (Gill, 1989) and such restrictions are released on the wintering grounds.

Larger‐scale patterns might also link to such individual behaviors. Newton (1995,2003) found that for Afro‐Palearctic landbirds, the species’ total winter ranges were on average smaller than breeding ranges. In the most extreme case, the lesser grey shrike *Lanius minor*, the breeding range is seven times larger (Herremans, 1998). In other species, the wintering range is larger than the breeding range but the wintering ranges are generally poorly known and may be overestimated, for example, if parts of the range are only occupied seasonally (Herremans, 1998). Because of the smaller average wintering ranges, densities must on average be higher on the African wintering grounds than during breeding (Newton, 2003) (presumably exaggerated soon after arrival by the larger number of extra young produced during breeding). Assuming that suitable area is occupied, more individuals can only coexist on a smaller area, if either (a) home ranges are smaller or (b) individual home ranges overlap more. The relationship between breeding and wintering might well vary among species according to variation in ecology and basic needs.

**Figure 1:**
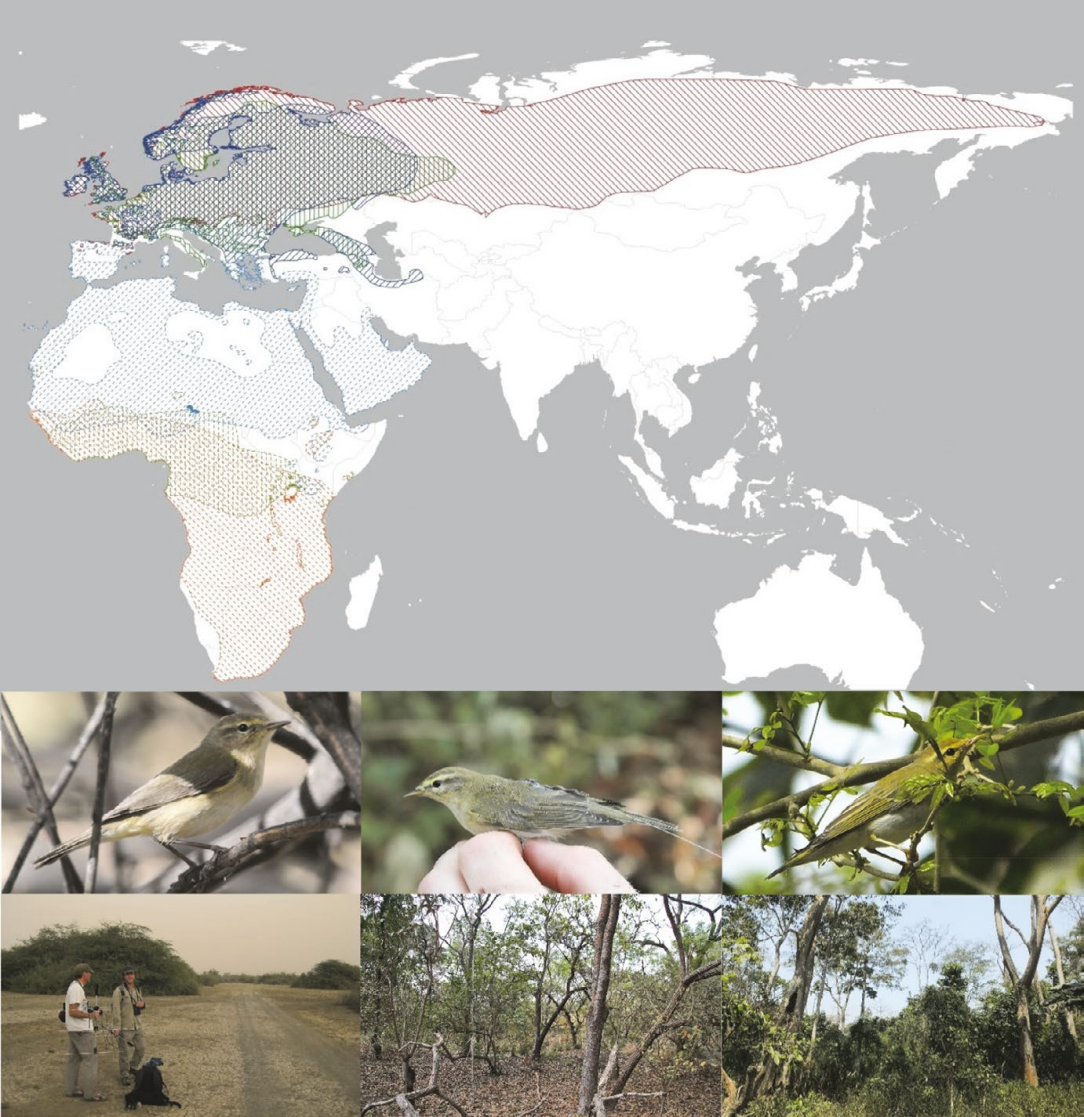
Breeding (darker colors and line texture) and wintering (paler color and dotted texture) distributions of chiffchaff (blue, insert below typical individual and winter study site habitat), willow warbler (red, middle), and wood warbler (green, right). Distribution of Siberian subspecies *tristis* of chiffchaff is removed because it is wintering in Asia. Data used with permission from BirdLife International and Handbook of the Birds of the World (2017)

We focus on three morphologically and ecologically similar species of *Phylloscopus* warblers, willow warbler *P. trochilus*, chiffchaff *P. collybita,* and wood warbler *P. sibilatrix* (Figure 1). They are all insectivorous foliage gleaners and feed on similar prey during the breeding season (Tiainen, 1982). Their breeding ranges overlap in Europe (Hagemeijer & Blair, 1997) but they separate into different climate zones in Africa during the wintering season (Urban, Fry, & Keith, 2002) where *Phylloscopus* species are sometimes observed in inter‐ as well as intraspecific flocks (Mallord et al., 2016; Salewski, Bairlein, & Leisler, 2002; Sorensen, 2014). Because of their similarity, we assume that differences in spatial and migratory behavior are primarily due to adaptations to the specific habitats that birds occupy in each season. Nonbreeding ranges are on average smaller than breeding ranges, and this was also the case in willow (15% smaller) and wood (6%) warblers but not in chiffchaffs (118% larger; Figure 1; Areas from distribution maps in BirdLife International & Handbook of the Birds of the World, 2017) which could, however, be affected by patchy habitat or seasonal occupation.

Even though the three species have overlapping breeding ranges, their habitat requirements differ. Chiffchaffs and wood warblers are considered more habitat specialists (Gregory et al., 2005), with chiffchaffs preferring deciduous, mixed old forest and in some areas coniferous forest and wood warblers preferring mixed, broadleaved, and old deciduous forests (Smart et al., 2007; Tiainen, Vickholm, Pakkala, Piiroinen, & Viroleinen, 1983), than the more generalist willow warblers breeding in various habitat types including more open and disturbed habitats (Gregory et al., 2005; Smart et al., 2007; Tiainen et al., 1983). The possibility of occurring in the same type of habitat in the breeding and wintering seasons differs between the three species: for willow and wood warbler, habitat structurally similar to the breeding habitat is found within their wintering range, whereas this is not so for chiffchaffs wintering in the northern Sahel. The openness of the habitat could potentially influence home range size in the *Phylloscopus* warblers as has been found in the common cuckoo *Cuculus canorus* (Williams, Willemoes, Klaassen, Strandberg, & Thorup, 2016); the three *Phylloscopus* warblers mostly forage in trees and bushes and could increase home range size as a response to reduced number of these.

Here, we use a comparative approach to identify the factors driving spatial behavior in these three closely related species. We aim to understand how space and habitat use differs between them and how it differs between breeding and wintering seasons to broaden our understanding of year‐round space and habitat use. Using radio‐telemetry data collected on breeding and wintering grounds, we compare among species home range size and overlap, and habitat use (based on tree cover), and investigate links for these parameters between seasons. Furthermore, we aim to broadly investigate whether smaller home range size and/or increasing overlap during wintering could be contributing to the decreasing total range size of willow and wood warblers in the wintering season and vice versa for chiffchaffs. It should be noted that even though breeding and wintering are likely to be within the overall areas used by the same populations, technical limitations prevent us from directly linking breeding and wintering individuals. As habitat associations often vary within a species geographical range and even among individuals, this restricts general inference.

## 2 METHODS

We radio‐tracked three *Phylloscopus* species during summer and winter: willow warbler (winter: *n* = 9, breeding: *n* = 13), wood warbler (*n* = 17, *n* = 14), and chiffchaff (*n* = 11, *n* = 7) to estimate space and habitat use. In breeding season, only males were tracked, and in winter, individuals could not be sexed; hence, probably both females and males were tracked. In the breeding season, all three species were tracked at deciduous forest sites in western Denmark (56.84°N, 10.24°E), and wood warblers also in eastern Denmark (56.07°N, 12.23°E). Willow warblers were tracked in 2013 and 2015, wood warblers were tracked in 2013 and 2014, and chiffchaffs were tracked in 2015. In winter, willow warblers were tracked at two sites in dry Savannah forest in Northern Ghana (9.09°N, 1.82°W), which is within the known wintering area of Danish breeding birds (Lerche‐Jørgensen, Willemoes, Tøttrup, Snell, & Thorup, 2017), while wood warblers were tracked at a site in the Guinea forest‐savannah transition zone in Southern Ghana (6.65°N, 0.7°W) in the same region as a Danish breeding bird wintering in Cotê d'Ivoire (Tøttrup, Pedersen, & Thorup, 2018). Chiffchaffs were tracked at a field site dominated by tamarisk *Tamarix sp*. in Djoudj National Park, Senegal (16.36°N, 16.26°W), where North European breeding birds have been reported (Wernham, 2002). Willow warblers were tracked in 2011 and 2012, wood warblers in 2012 and 2013, and chiffchaffs in 2012.

The birds were trapped in mist nets with or without the use of playback of birds’ calls or songs. It is unlikely that all conspecifics within the study area were captured but we consider the trapped birds to constitute a near‐random sample of birds in the study areas. As the estimated home ranges overlapped at all sites, our procedures were catching birds from a broad range within the study area both on breeding and wintering grounds and irrespective of whether birds were territorial or not.

The trapped birds were fitted with ~0.3 g radio tags (Holohil system Ltd or PicoPip tag, Biotrack Ltd), either sewn onto a little piece of fabric and glued to the back of the bird, on an area of shaved skin, using eyelash adhesive (eyelash adhesive was used to make sure that the tag would fall off before the birds initiated spring migration), or glued to the two central tail feathers with super glue and tied with dental floss (wood warblers only in winter). Transmitter life varied—in some individuals, no positions were obtained after release and others were tracked for up to nearly three weeks. If an otherwise active transmitter was not detected, it was searched for intensively in the surrounding area. We used handheld VHF receiver (for the Holohil tags) and Telonics TR‐4 receiver (for the Biotrack tags) and three‐element Yagi antennas to track the birds. At least one position a day was obtained per bird; if more were obtained, we kept a minimum time gap of one hour between positions to reduce spatial autocorrelation. On average, 29 ± 13 (mean ± *SD*) positions per individual were included (Chiffchaff winter: 31 ± 6; breeding: 25 ± 4. Willow warbler winter: 12 ± 1; breeding: 30 ± 9. Wood warbler winter: 34 ± 16; breeding: 32 ± 13).

Linear and mixed models were fitted in SAS 9.4. Spatial analyses were carried out in R (R Core Team, 2016) using the AdehabitatHR package (Calenge, 2006). For individuals with at least 10 positions, we calculated home range sizes with 50% and 100% minimum convex polygons (MCP) and 50% and 90% kernel utilization distribution (KUD). For the KUDs, we used the smoothing factors “href” because this is considered a more conservative estimate than “LSCV” (Börger et al., 2006). We used the nlme package (Pinheiro, Bates, DebRoy, & Sarkar, 2017) to test for a difference in home range size between species and season with a mixed linear model as suggested by Börger et al. (2006) where the number of points was included as a random factor and home range size was log‐transformed. We only report the results from 90 KUDs in the result section as these are considered reliable (Börger et al., 2006); home range estimated as 50% KUD, 50% MCP, and 100% MCP can be found in Figure S1.

To test for differences in degree of space sharing, we calculated home range overlap. The maximum distance at which overlap occurred was 427 m between two individuals’ home range centroids. Therefore, overlap was only estimated for individuals within that distance. Furthermore, we only estimated the overlap if the time period in which two individuals had been tracked overlapped. To take into account that not all individuals within that range were likely to be caught, we base our results on average overlap between pairs of individuals which is not in itself biased by missing individuals in the sample as long as the sample of individuals within the range can be considered a random sample. Additionally, the data were collected in the same way across species and seasons making them comparable, and any differences between groups are likely reflecting true differences. Because we were mainly interested in finding out whether the individuals were likely to be in the same areas at the same time, we used overlap indices that take nonuniform space use into account. Home range was calculated with the 90% kernel utilization distribution and smoothing factor “href” as for home range size calculations. We used the indices, probability home range (PHR) overlap and Utilization Distribution Overlap Index (UDOI) as recommended by Fieberg and Kochanny (2005). PHR_ij_ calculates the volume of animal *j*'s home range inside animal *i*'s, that is, estimates the probability of animal *j* being located in animal *i*'s home range, and the index ranges from 0 to 1. The UD overlap index (UDOI) is the joint distribution of two animals. It is 0 with no overlap and 1 with 100% overlap, and the two birds have uniform space use but can be >1 if there is a large degree of overlap of nonuniform space use. We used a linear model to test whether the overlap differed between species and season. In winter, where the overlap was considerable in all three species, we tested if home range overlap increased with increasing home range size using a mixed linear model with species as random factor.

To investigate whether there was a difference in habitat structure between breeding and winter and whether the habitat structure used differed between the three species, we used the dataset *treecover2000* (Hansen et al., 2013) which consists of the percentage canopy closure for all vegetation higher than 5 m inside 30 × 30 m grid cells. We extracted the mean tree cover inside the 90 KUD home ranges (tree cover inside 50% KUD, 50% MCP, 100% MCP see Figure S2). We used a linear model to test for an effect of species and season. Further, we tested whether home range size was correlated with tree cover, for breeding and winter separately, with a linear mixed model with log‐transformed home range size and species as random factor.

## 3 RESULTS

**Figure 2:**
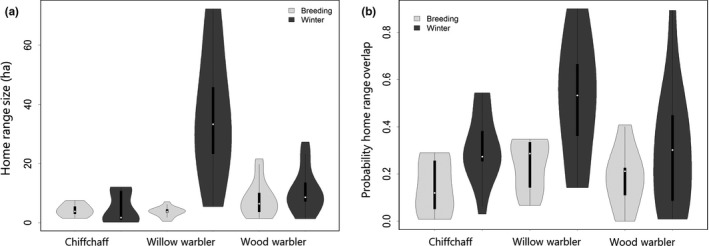
Violin plots showing (a) home range size calculated with 90% KUDs and (b) probability home range overlap (based on each individual's average overlap with other individuals calculated using 90 KUDs). Whiskers in violin plots indicate 95% confidence intervals; boxplots inside show median and quantiles

**Figure 3:**
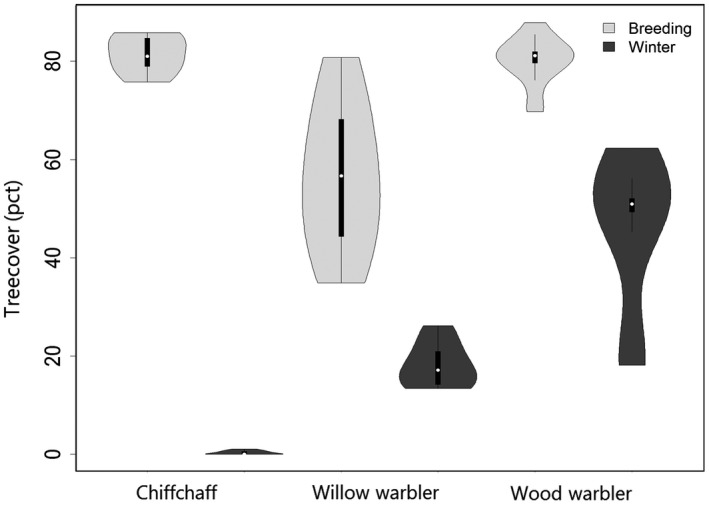
Violin plots showing tree cover inside 90% KUDs. Whiskers in violin plots indicate 95% confidence intervals; boxplots inside show median and quantiles

Home ranges were larger in winter than in the breeding season for willow warbler (Effect of season in Mixed Model; *df* = 1, *F* = 9.06, *p* = 0.0037), and the home ranges varied among species (Effect of species; *df* = 2, *F* = 8.95, *p* = 0.0004) with the willow warbler's winter home range being larger than that of the others (Figure 2a, Table 1). Home range size varied less during the breeding season (Figure 3a, Table 1).

**Table 1:** Mean estimates of home range size, overlaps, and tree cover inside home ranges

Species	Season	Home range size (ha)		Home range overlap PHR		Home range overlap UDOI		Tree cover (%)	
		Mean	*SE*	Mean	*SE*	Mean	*SE*	Mean	*SE*
Chiffchaff	Breeding	7.88	9.66	0.16	0.05	0.04	0.03	81.43	3.92
	Winter	10.60	12.59	0.31	0.06	0.12	0.04	0.31	5.01
Willow warbler	Breeding	3.62	9.78	0.31	0.08	0.12	0.08	56.08	4.86
	Winter	54.37	13.55	0.53	0.12	0.24	0.07	17.73	6.73
Wood warbler	Breeding	4.23	7.89	0.15	0.08	0.06	0.05	80.12	4.80
	Winter	4.90	10.09	0.36	0.60	0.19	0.06	45.42	6.26

Note

Home range test estimates refer to back transformed values of the model estimates from the linear mixed model testing for differences between species and season of the log‐transformed home range size.

Home range overlap was significantly higher in winter than in breeding season based on PHR (Effect of season in Mixed Model; *df* = 1,191, *F* = 14.79, *p* = 0.0002; Figure 2b) across the three species. The probability overlap varied among species (*df* = 2,191, *F* = 4.62, *p* = 0.0110) and was higher in willow warblers than for the other two species (Figure 2b). There was high individual variation for all species (Figure 2b). The UDOI overlap was also overall higher in winter than in the breeding season (Effect of season in Mixed Model; *df* = 1,191, *F* = 16.10, *p* < 0.0001) but was always <100%.

Home range overlap was not significantly correlated with home range size (estimate = −0.06, *SE* = 0.03, *df *= 30, *t* = −1.74, *p* = 0.09).

The home range size was not correlated with tree cover inside home ranges in summer (estimate = −0.0004, *SE *= 0.01, *t* = −0.03, *p* = 0.97) nor in winter (estimate = 0.00005, *SE* = 0.02, *t* = 0.003, *p* = 1). The percentage tree cover inside the home range was significantly higher in breeding than in wintering season (Effect of season in Linear Model; *df* = 1, *F* = 400.36 *p* < 0.0001). Tree cover differed between the three species (Effect of species in Linear Model; *df* = 2, *F* = 46.77, *p* < 0.0001). In winter, it was highest for the wood warbler and lowest for chiffchaff (Figure 3). In the breeding season, the tree cover was lowest for willow warbler and similar between the other two (Figure 3).

## 4 DISCUSSION

We identified differences in space and habitat use of three migratory species between both ends of the migratory flyway. Willow warblers increased home range size in winter compared to breeding season in contrast to chiffchaffs and wood warblers that maintained the same home range size between seasons. In winter, willow warblers’ home range sizes were larger than for the other two species. The overlap between home ranges was larger in winter than in breeding season, where larger overlaps in willow warblers were indicated. Tree cover differed between winter and breeding season for all species but most markedly for chiffchaff. Home range size seemed not to be related to tree cover inside home ranges.

We found a general pattern of larger home range overlap in winter which may allow smaller total species winter ranges and potentially allowing the expected higher densities in winter in willow and wood warblers. In contrast, home range sizes were similar or larger. That home range overlap during winter was considerable in all three species indicates a general lack of territoriality, in line with observations from the wintering area (Mallord et al., 2016; Sorensen, 2014; Willemoes et al., 2017). The very large wintering range of chiffchaffs is not in accordance with our observations of larger home range overlap also in this species. The large estimated nonbreeding range of chiffchaffs includes most of the Sahara, and within large parts of the estimated distribution, suitable areas are likely far between resulting in a much smaller area actually used within the extent of the whole wintering range.

Only willow warblers had larger home ranges in winter. Given that they often forage in flocks during winter (Sorensen, 2014; Willemoes et al., 2017), the use of a larger area than when they are territorial during breeding is perhaps not surprising. However, the increase in willow warblers’ home range size and not in the other two species indicates that resource abundance or the ability to exploit resources in winter and breeding differs between the three species. In willow warblers, being generalist, birds could potentially occur in a broader range of habitat (Gregory et al., 2005; Kassen, 2002) which means that they, compared to the specialists, chiffchaff and wood warbler, are able to exploit more patches in the open and degraded habitat that migrants are often found in (Jones, Salewski, Vickery, & Mapaure, 2010; Wilson & Cresswell, 2006).

As a generalist, we expected that willow warblers would be less territorial (Feinsinger & Colwell, 1978; Kassen, 2002). However, the home range overlap was only slightly and nonsignificantly larger in willow warbler than in the other two species. None of the individuals showed overlap values larger than could be expected by uniform space use (if UDOI > 1) (Fieberg & Kochanny, 2005). This could either indicate that intraspecific flocks are temporary group structures (as indicated in Sorensen, 2014) or that we simply did not track more than one bird from same flock.

Contrary to expectations, individuals wintering in more open habitat did not have larger home ranges, despite the reduction in tree cover from breeding to wintering seasons and the large variation in tree cover between species. Such a pattern was demonstrated in common cuckoos that had smaller home ranges in areas with dense forest cover, presumably directly related to habitat suitability (Williams et al., 2016). The tree cover used for the analysis was tree cover from trees taller than five meter, and this might suggest that the birds forage in lower vegetation in winter, which was indicated in a study of chiffchaffs in the winter area where they found the stomach content contained geophile species (Abdeljalil, Daoudi‐Hacini, & Doumandji, 2016). However, it does not seem to be the case in willow warblers and wood warblers which at the wintering ground are found to prefer trees taller than average (Mallord et al., 2016; Willemoes et al., 2017) and the tree cover we find is in line with the tree cover reported from a ground‐based survey (Mallord et al., 2016). Thus, our findings suggest that wintering habitat for the three species was generally more open, and for chiffchaff maybe shorter vegetation, than during breeding. This indicates that at least on a finer scale, migrants use different habitat in winter compared to breeding in contrast to the general assumption of similar habitat use (Newton, 2008; Sheehan & Sanderson, 2012).

We acknowledge that tracking the same individuals in both seasons would have been the most appropriate way to assess the relationship between breeding and wintering behavior and habitat but, given the small size of the species involved, the lack of adequate miniaturization of technology prevents this. At least on a broad scale, the wintering areas of tracked birds appear to encompass that occupied by the studied breeding populations as confirmed by both geolocator and ringing data (Lerche‐Jørgensen et al., 2017; Tøttrup, Pedersen, & Thorup, 2018; Wernham, 2002). Further, breeding habitat across large areas of the breeding distribution is similar to that in Denmark (e.g., Sweden and England; Grell, 1998; Larsson, 2001; Smart et al., 2007).

We find that space and habitat use changes between breeding and winter. Though the three *Phylloscopus* species are observed in flocks on the wintering grounds, chiffchaff and wood warbler did not increase home range size in winter which indicates that suitable habitat might limit the area use of specialists or that higher food abundance in wood warbler's and chiffchaff's habitat allows them to maintain same home range size as during breeding season. Furthermore, home range size did not increase with decreasing tree cover, and generally tree cover was much lower in wintering than in breeding. This indicates that an otherwise common assumption of niche‐tracking might not apply on a fine scale. Tree felling and destruction of natural habitat are widespread throughout Africa. Given the lower wintering range tree cover, moderate tree felling might not affect the area of suitable habitat negatively (Mallord et al., 2018). Nevertheless, sustainable land use will likely be important to avoid declining wintering area for migrants, potentially contributing to future declines in Afro‐Palearctic migrants.

## CONFLICT OF INTEREST

None declared.

## AUTHOR CONTRIBUTIONS

KT conceived the study. KT, MLJ, MW and APT designed the study. All authors contributed to the fieldwork. MLJ, MW and KT analyzed the data. All authors interpreted the results. KT and MLJ wrote the manuscript; other authors provided editorial advice.

## DATA AVAILABILITY

The data for this study are included as supplementary information.

## APPENDIX 1 Individual data on radio‐tracked individuals included in the study

**Table:**

Bird_ID	Species	Season	# locations	KD90_ha	Treecover	# ind overlap
1	Chiffchaff	Winter	37	12.07	1.09	10
2	Chiffchaff	Winter	39	2.84	0.41	10
3	Chiffchaff	Winter	36	11.76	0.64	10
4	Chiffchaff	Winter	18	10.27	0.52	10
5	Chiffchaff	Winter	32	11.16	0.80	10
7	Chiffchaff	Winter	32	0.23	0	10
8	Chiffchaff	Winter	31	1.55	0	10
9	Chiffchaff	Winter	33	0.15	0	10
10	Chiffchaff	Winter	27	1.63	0	10
11	Chiffchaff	Winter	27	1.71	0	10
12	Chiffchaff	Winter	26	0.53	0	10
13	Willow	Winter	12	23.73	14.16	2
14	Willow	Winter	13	26.48	16.55	2
15	Willow	Winter	11	60.05	14.25	2
18	Willow	Winter	11	41.03	23.00	
20	Willow	Winter	11	72.29	20.20	1
21	Willow	Winter	11	5.42	26.19	1
22	Willow	Winter	11	198.32	14.08	2
23	Willow	Winter	12	40.10	17.76	2
24	Willow	Winter	15	21.86	13.38	2
26	Wood	Winter	16	8.70	51.04	5
27	Wood	Winter	11	8.20	49.61	5
30	Wood	Winter	45	1.70	59.86	
31	Wood	Winter	42	8.93	52.04	5
32	Wood	Winter	29	22.77	51.75	5
33	Wood	Winter	24	13.64	49.34	5
34	Wood	Winter	31	11.02	50.67	5
35	Wood	Winter	66	7.14	19.14	1
36	Wood	Winter	47	27.32	58.20	1
38	Wood	Winter	28	8.60	57.72	1
39	Wood	Winter	29	22.65	18.10	1
40	Wood	Winter	50	1.35	20.72	2
41	Wood	Winter	41	13.55	19.02	1
43	Wood	Winter	55	7.88	50.30	
44	Wood	Winter	11	3.53	50.93	1
45	Wood	Winter	43	8.24	51.39	1
46	Wood	Winter	11	5.03	62.33	
G01	Chiffchaff	Breeding	26	3.76	84.71	5
G03	Chiffchaff	Breeding	21	1.47	85.82	2
G04	Chiffchaff	Breeding	29	7.50	78.01	5
G05	Chiffchaff	Breeding	26	3.75	75.78	6
G06	Chiffchaff	Breeding	19	2.15	84.67	6
G08	Chiffchaff	Breeding	29	6.23	81.04	5
G09	Chiffchaff	Breeding	28	4.74	79.95	5
14	Willow	Breeding	25	3.71	68.64	1
15	Willow	Breeding	31	0.48	68.17	4
16	Willow	Breeding	41	1.37	48.29	4
17	Willow	Breeding	26	3.40	43.73	4
18	Willow	Breeding	43	2.06	44.33	4
19	Willow	Breeding	43	3.52	34.85	4
42	Willow	Breeding	34	3.62	56.67	2
43	Willow	Breeding	22	4.02	35.77	
45	Willow	Breeding	31	3.96	57.76	2
46	Willow	Breeding	26	4.71	51.51	2
48	Willow	Breeding	33	4.66	61.80	1
L02	Willow	Breeding	27	4.52	76.72	1
L10	Willow	Breeding	11	7.04	80.76	1
a01	Wood	Breeding	12	18.71	77.64	1
a02	Wood	Breeding	47	10.64	69.73	1
a03	Wood	Breeding	39	9.79	70.74	
a04	Wood	Breeding	18	4.91	79.87	
a09	Wood	Breeding	36	1.68	81.79	
a10	Wood	Breeding	24	3.19	87.88	
A11	Wood	Breeding	16	1.41	85.38	
A12	Wood	Breeding	22	10.17	79.59	
B111	Wood	Breeding	40	21.56	81.01	4
B113	Wood	Breeding	44	5.15	80.52	4
B120	Wood	Breeding	45	7.31	82.43	4
B121	Wood	Breeding	42	5.42	82.05	4
B147	Wood	Breeding	46	7.43	81.29	
B149	Wood	Breeding	14	2.91	81.75	4
