# Supplementary material for: Spatial behavior and habitat use in widely separated breeding and wintering distributions across three species of long‐distance migrant Phylloscopus warblers
Source: Ecol Evol. 2019 May 24;9(11):6492–500. doi: 10.1002/ece3.5226 (PMC6580274; doi:10.1002/ece3.5226)
Supplement: Supplementary file 1 [file ECE3-9-6492-s001.docx]

**Supplementary Information**


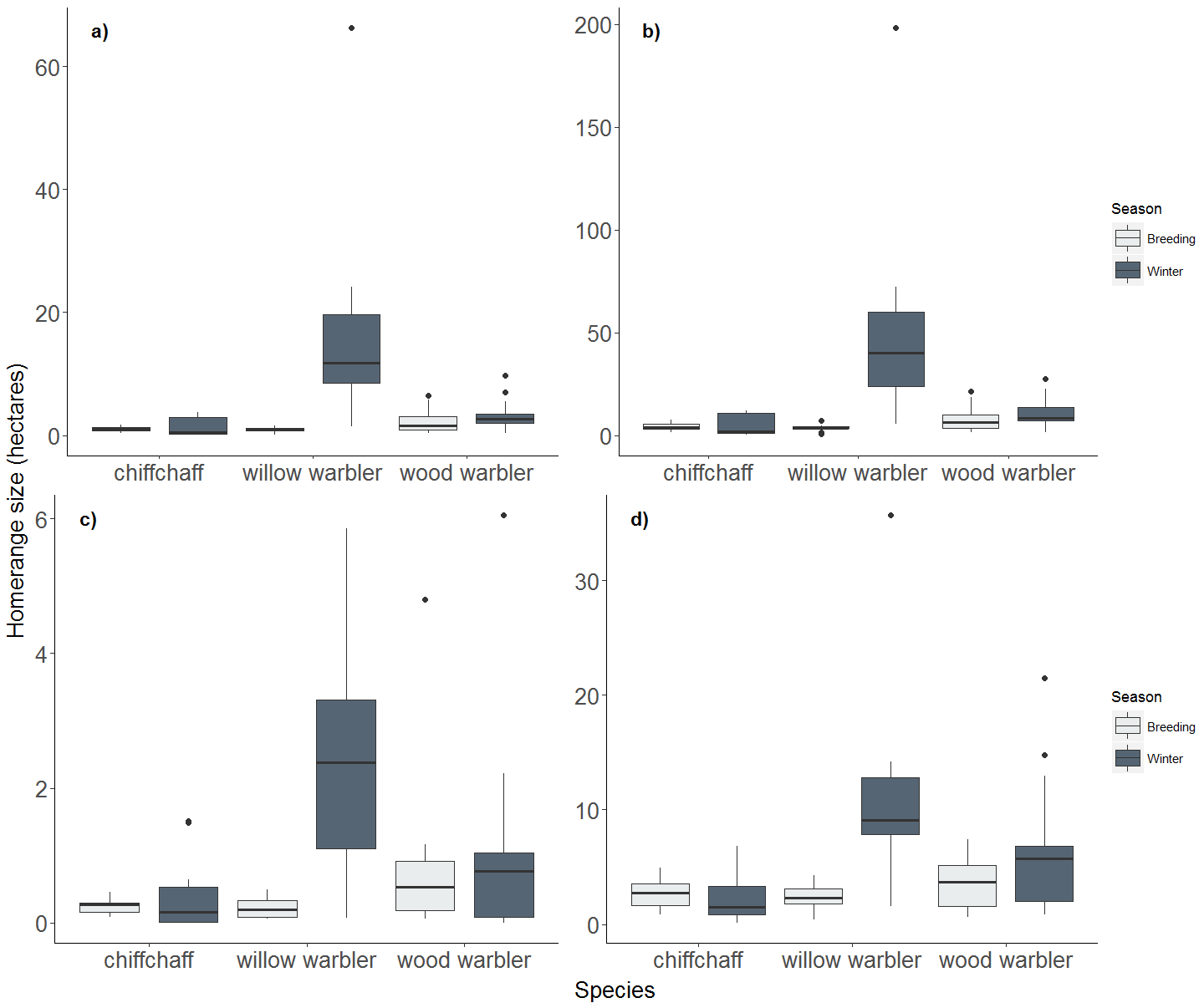


Figure S1. Home range sizes calculated as a) 50 % Kernel densities, b) 90 % Kernel densities, c) 50 % Minimum Convex Polygons and d) 100 % Minimum Convex Polygons.


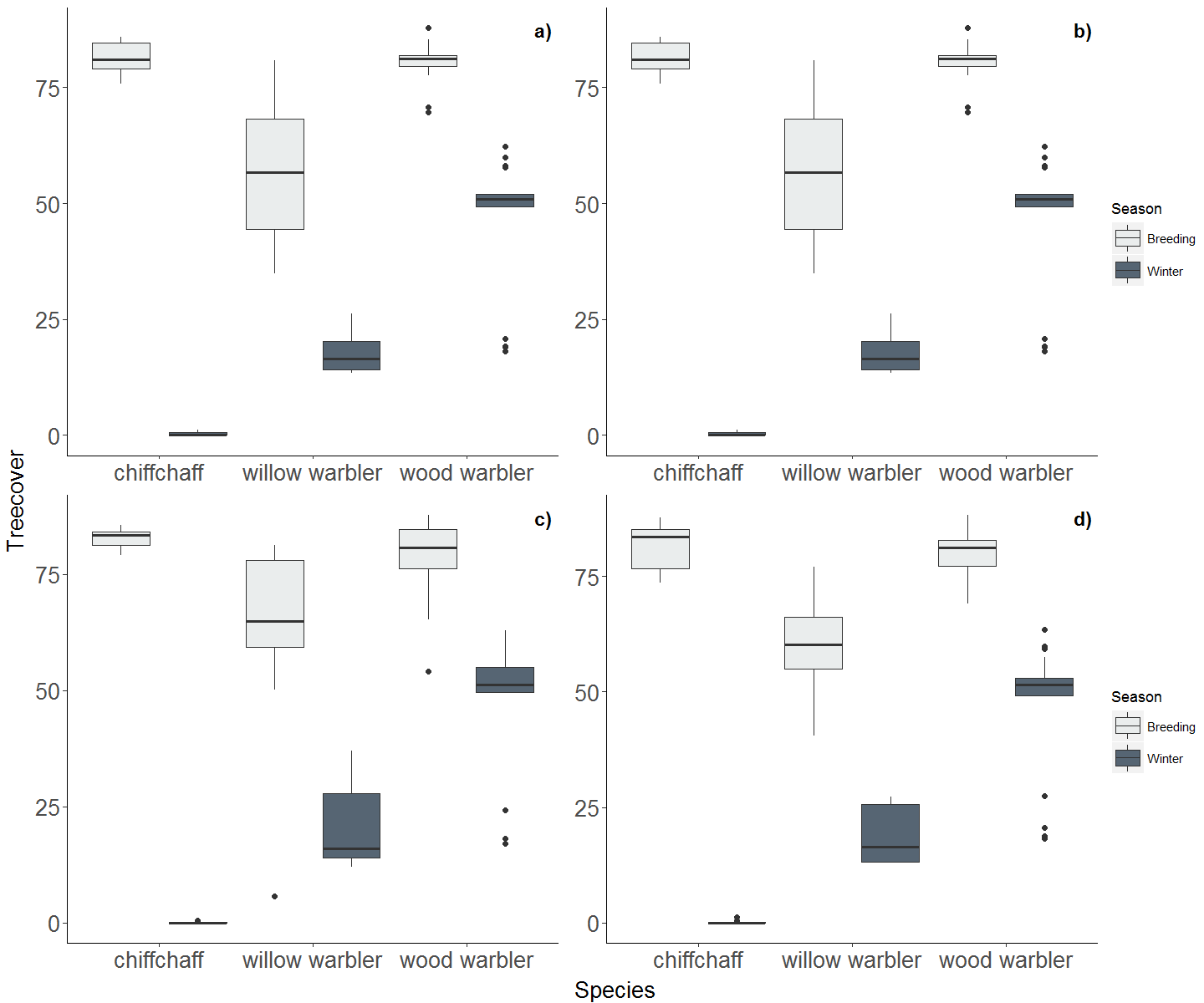


Figure S2. Percentage of tree cover inside a) 50 % Kernel densities, b) 90 % Kernel densities, c) 50 % Minimum Convex Polygons and d) 100 % Minimum Convex Polygons.
